# Scrofuloderma, an Old Acquaintance: A Case Report and Literature Review

**DOI:** 10.3390/idr17040096

**Published:** 2025-08-06

**Authors:** Heiler Lozada-Ramos, Jorge Enrique Daza-Arana

**Affiliations:** 1 Medicine Program, Faculty of Health, Universidad Santiago de Cali, Palmira 763532, Colombia; 2 Doctoral Program in Infectious Diseases, Universidad de Santander—UDES, Bucaramanga 680003, Colombia; 3 Physiotherapy Program, Faculty of Health, Universidad Santiago de Cali, Cali 760035, Colombia; jorge.daza01@usc.edu.co

**Keywords:** cutaneous tuberculosis, extrapulmonary tuberculosis, scrofuloderma, scrofula, BCG

## Abstract

Scrofuloderma, a cutaneous manifestation of tuberculosis, is a rare but clinically significant form of mycobacterial infection. It typically results from the local spread of Mycobacterium tuberculosis from an infected lymph node or bone area to the overlying skin. This disease is mainly characterized by chronic granulomatous inflammation, leading to skin ulcers and abscesses. Due to its nonspecific clinical presentation, scrofuloderma can mimic various dermatological conditions, making its diagnosis particularly challenging. This case report presents the clinical course of a patient who was positive for the Human Immunodeficiency Virus (HIV) with a diagnosis of scrofuloderma, managed at a tertiary healthcare center, with follow-up before and after treatment. A literature review was also made, highlighting the importance of maintaining a high index of clinical suspicion and utilizing appropriate diagnostic methods to ensure timely diagnosis.

## 1. Introduction

In 2022, an estimated 10.6 million people (IU: 9.9–11.4 million) worldwide developed tuberculosis (TB), with a global incidence rate of 133 cases (IU: 124–143) per 100,000 individuals. Over the past years, TB has remained among the top 10 leading causes of mortality worldwide [1]. TB is caused by Mycobacterium tuberculosis (Mtb), a member of the Mtb complex, which is transmitted via aerosols generated by individuals with active lung disease [2]. It has been estimated that 5–10% of individuals with latent TB will develop active TB, promoted by factors such as diabetes, kidney failure, HIV/AIDS (Acquired Immune Deficiency Syndrome), cancer, and co-infections such as COVID-19 [3].

Extrapulmonary TB (EPTB) accounts for approximately 15–20% of all TB cases. Unfortunately, TB can mimic a wide variety of conditions and should always be considered in subjects with diabetes, immunodeficiency syndromes (such as AIDS), or those receiving immunosuppressive therapies such as chemotherapy. Even though EPTB can involve practically all anatomical sites, the most frequently involved include the lymph nodes (50%), pleura (18%), genitourinary system (13%), bones and joints (6%), gastrointestinal system (6%), central nervous system (3%), and spine (3%) [4,5]. Cutaneous TB (CTB) is a rare form of EPTB, representing <1–2% of all TB cases [6]. One of its presentations is scrofuloderma, also known as TB colliquativa cutis. Scrofuloderma arises from the direct extension of infection from an underlying focus, commonly involving lymph nodes, bones, joints, testes, or the epidermis. The most frequent sites of scrofuloderma onset are the thorax, neck, and armpit. Clinically, it presents as violaceous, firm, painless subcutaneous nodules that tend to suppurate, forming ulcers and sinus tracts with serous, purulent, or caseous material drainage. CTB has a wide range of morphologic presentations and may mimic several infectious and noninfectious dermatoses, so clinical diagnosis is often delayed and requires a high index of suspicion. Therefore, in the presence of regional lymphadenopathy, a possible systemic origin of tuberculosis should be evaluated [7].

The emergence of multidrug-resistant Mtb strains challenges the prognosis and management of TB, underscoring the importance of recognizing all forms of the disease, including the cutaneous manifestations. Although epidemiological data on scrofuloderma prevalence are limited, case reports emphasize the need for heightened clinical suspicion and awareness [8]. In immunosuppressed individuals, the prognosis is variable, depending on nutritional support, early diagnosis, and surveillance and monitoring of adverse events and drug interactions, as well as the degree of immunosuppression [9]. However, due to the frequent association with pulmonary TB or other forms of EPTB, early diagnosis and appropriate treatment are crucial to avoid serious complications or even death [10]. Differential diagnoses include pyoderma gangrenosum, subcutaneous fungal infections, syphilitic gumma, and actinomycosis.

In this paper, we describe the clinical case of a young HIV-positive patient with scrofuloderma and concomitant pulmonary TB who was timely diagnosed and treated, resulting in the resolution of active TB. A literature review on scrofuloderma, a rare but current form of EPTB, is also presented. Even if cases of scrofuloderma have been reported, there are few reviews on the topic. Furthermore, published cases lack photographic evidence before and after treatment.

## 2. Case Presentation

**Figure 1:**
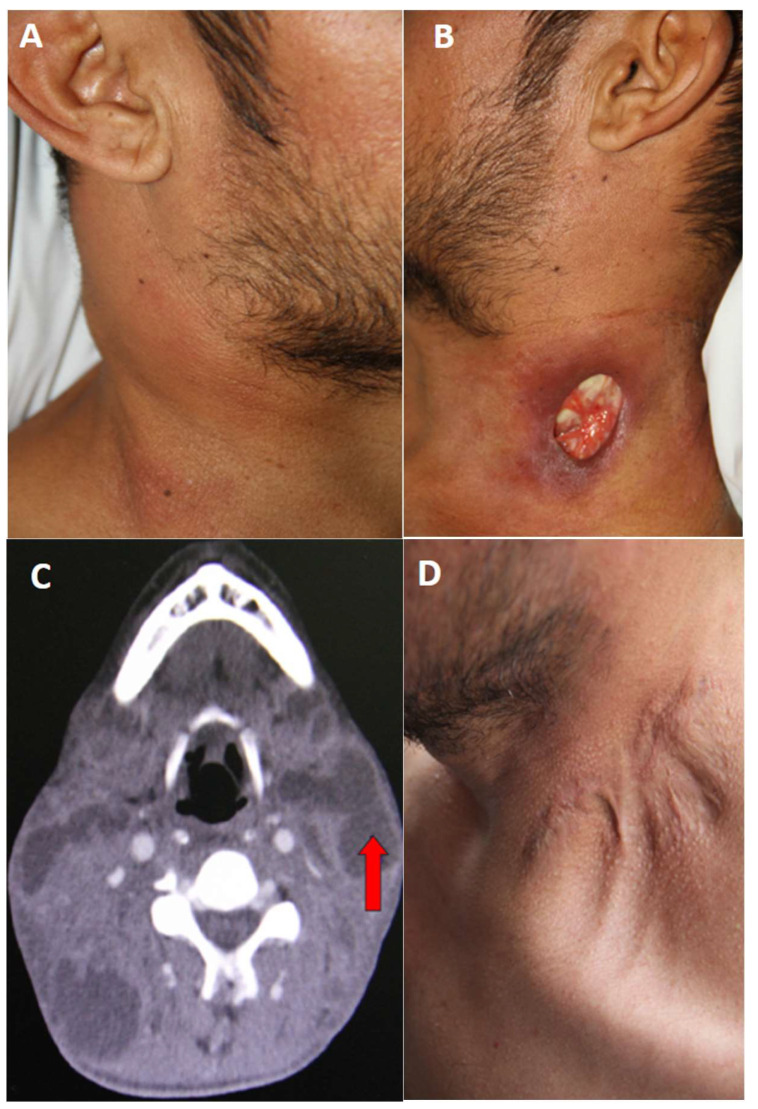
(**A**) Presence of multiple adenopathies on the right side of the neck. (**B**) Ulcer at the base of the left side of the neck, with well-defined, hyperpigmented borders. Caseous material is observed in the background. (**C**) Computed tomography scan of the neck showing multiple bilateral adenopathies (red arrow). (**D**) Scar on the left side of the neck, with evidence of retractions and keloid after completion of anti-tuberculosis treatment.

Here we report a case of a 41-year-old man with a history of smoking and de novo HIV infection. The patient had no history of diabetes mellitus or known contact with individuals exhibiting respiratory symptoms. His symptoms began 2 months earlier with odynophagia, decreased appetite, fever, chills, sweating, and 7 kg weight loss. In the ensuing weeks, he developed cervical masses, initially on the right side and subsequently on the left. The following month, the patient noted the appearance of an oval-shaped ulcer at the base of the left side of his neck (left supraclavicular region), exuding a whitish, lumpy material, with progressive enlargement of the ulcer. Upon hospitalization, vital signs were within normal limits except for a fever of 38.4 °C. Physical examination revealed multiple mobile, nonadherent, nontender, and nonfluctuant cervical bi-sided lymphadenopathies (Figure 1A). No adenopathies were found in any other region of the body. Examination of the left cervical region showed a 3 × 2 cm ulcerative lesion with well-defined hyperpigmented margins and whitish material at its base (Figure 1B). Samples from the ulcer were collected for bacteria and mycobacteria, as well as bacilloscopy testing.

Laboratory results revealed a leukocyte count of 12,200 leukocytes/mm^3^ (68% neutrophils), hemoglobin level of 10.5 g/dL, normal platelet count, and creatinine of 1.1 mg/dL. Liver function test results and serum electrolytes were within normal ranges. Chest X-ray showed only mild bibasal interstitial infiltrate and no calcifications, pleural effusions, or cavitary lesions. A presumptive diagnosis of nodal TB with scrofuloderma, de novo HIV infection, and possible lymphoproliferative disease was considered. Bacterial culture of cervical ulcer discharge was negative for bacteria, but bacilloscopy reported the presence of +++ acid-alcohol-resistant bacilli (AARB), BK in sputum with +++ AARB, and a serum ADA (Adenosine Deaminase) test of 27.1 U/L (0–15 IU/L). Cervical computed tomography demonstrated bilateral adenopathy (Figure 1C). Immunological studies revealed a CD4 cell count of 39 cells/microliter; the viral load was 341 275 copies/mL.

Three weeks later, mycobacterial liquid medium cultures were obtained from the skin lesion, and the cervical lymph node aspirate grew Mtb. The patient was initiated on conventional treatment for lymph node and pulmonary TB. The regimen consisted of daily isoniazid, rifampicin, ethambutol, and pyrazinamide for the first 2 months, followed by twice-weekly isoniazid and rifampicin for an additional 4 months. Antiretroviral treatment was initiated 3 weeks after the start of TB treatment. During outpatient follow-up, regression of the skin alterations was observed 3 months after starting treatment, with resolution of the neck lesion and absence of lymphadenopathies at the end of the treatment (Figure 1D).

## 3. Discussion

The WHO’s main TB directed is the End TB Strategy, which has the ambitious goals of 90% reduction in TB incidence and 95% reduction in TB deaths by 2035 [11]. A critical component of these efforts will be widespread implementation of TB preventive therapy, especially among TB case contacts, children, and immunosuppressed individuals. Therefore, timely diagnosis and treatment of pulmonary and extrapulmonary of TB forms is essential.

Scrofula (King’s evil) refers to cervical tuberculous lymphadenopathy, an antiquated term for tuberculous infection of the lymph nodes of the neck [12], while scrofuloderma or TB colliquativa cutis represents an endogenous cutaneous manifestation of TB. It is characterized by the development of a subcutaneous nodule overlying an infected lymph node, joint, bone, or epididymis, which subsequently ruptures, forming an undermined ulcer with granulation tissue at its base. The most affected sites include the neck, axilla, and preauricular, submandibular, occipital, and inguinal regions. Scrofuloderma could be the first clinical manifestation of EPTB [13,14]. The same occurred in the patient, where the first manifestation of EPTB was in the cervical lymph nodes.

While there are different classification systems for CTB, the most widely used to cover all variants include the following: exogenous CTB (tuberculous chancre and TB verrucosa cutis), endogenous CTB related to contiguity or autoinoculation (scrofuloderma, TB cutis orificialis, some cases of lupus vulgaris), hematogenous dissemination (lupus vulgaris, tuberculous gumma, and acute miliary TB), tuberculids (papulonecrotic tuberculids, lichen scrofulous) and CTB secondary to bacille Calmette–Guérin (BCG) vaccine [9]. Scrofuloderma belongs to the EPTB group, which accounts for approximately 20% of all TB and is strongly associated with HIV infection, the emergence of multidrug-resistant TB, and immunosuppressive treatments [15]. In our case, scrofuloderma was associated with HIV infection.

Among the various clinical manifestations of CTB, scrofuloderma is one of the most frequent, accounting for 14.3–50.7% [15,16,17,18]. The clinical manifestations of scrofuloderma usually begin with adenopathies, which are frequently painless and mobile and occur as multiple or solitary lesions, in most cases with involvement at the cervical level. These appear as mobile subcutaneous nodules that soften to form painless, fluctuant abscesses. Subsequently, the overlying skin breaks down, resulting in depressed ulcers with irregular, linear, and dusky borders, a yellowish granular base, and fistulous tracts discharging purulent material. Finally, healing leads to the formation of retractile scars or keloids, as observed in the present case (Figure 1D) [19]. Despite advances in the diagnosis and treatment of TB, scrofuloderma remains difficult to diagnose due to its rarity and nonspecific clinical presentation.

Scrofuloderma can affect individuals of any age but is more frequently seen in children, adolescents, and elderly adults, who may have diminished immunological defenses. It may occur as an isolated cutaneous manifestation or in association with pulmonary or disseminated forms of TB. A cohort study by Mann et al. identified 15 patients with CTB and a positive serological status for HIV, in which the most prevalent manifestation was scrofuloderma (80%), with an average CD4+ cell count of 262 cells/µL [20]. Nevertheless, some studies have shown the association between HIV-positive patients with CD4+ counts <100 and the presence of EPTB [21]. Scrofuloderma is one of the few subtypes of CTB that causes systemic manifestations such as fever, weight loss, and respiratory symptoms such as productive cough [22].

The differential diagnosis of scrofuloderma includes atypical mycobacterial infections, sarcoidosis, vulgar warts, blastomycosis, botryomycosis, nocardiosis, sporotrichosis, paracoccidioidomycosis, lymphogranuloma venereum, hidradenitis suppurativa, leprosy, and tertiary syphilis, among others [19]. Atypical mycobacterial infections, such as *Mycobacterium avium* or *M. marinum*, cause chronic cutaneous lesions without systemic involvement and require molecular testing for proper identification. Sarcoidosis, though it also produces granulomas, is characterized by the absence of caseating necrosis and the presence of non-infectious granulomas in organs such as the lungs. Deep fungal infections, such as blastomycosis and paracoccidioidomycosis, produce ulcerated or verrucous lesions but are often accompanied by pulmonary or mucosal involvement and are diagnosed by culture or identification of characteristic yeast forms. Sporotrichosis can mimic cutaneous tuberculosis by forming ulcerated nodules along lymphatic channels, but it is typically associated with exposure to plant material and responds to antifungal therapy. Botryomycosis, a chronic bacterial infection, forms subcutaneous masses with purulent drainage and visible granules, like nocardiosis, which also presents with fistulous tracts and filamentous organisms on Gram staining. Sexually transmitted diseases such as lymphogranuloma venereum present with genital ulcers and suppurative inguinal lymphadenopathy, while tertiary syphilis may cause gummas—destructive granulomatous lesions with positive serology. Leprosy, in contrast, is distinguished by peripheral nerve involvement and hypopigmented, anesthetic skin lesions. Finally, hidradenitis suppurativa is a chronic inflammatory condition affecting apocrine gland-bearing areas such as the axillae and groin, characterized by abscesses and fistulas without systemic infection or the presence of bacilli in the lesions.

Depending on prior BCG vaccination status, the purified protein derivative skin test (tuberculin or Mantoux test) may yield a positive result. Similarly, cases have been reported on the occurrence of scrofuloderma after BCG vaccination [23]. BCG has a specificity of 63% and a sensitivity between 33% and 96% for CTB in general. Interferon-gamma has a sensitivity of 92% and a specificity of 76% in individuals with CTB [24]. It is essential to perform a chest X-ray to evaluate concomitant pulmonary TB. The majority of extrapulmonary TB has a primary focus on the lung, and from this focus, there is lymphatic spread to the peripheral lymph nodes. This happened in our case, where pulmonary involvement was confirmed with the performance of bacilloscopy.

Histopathology examination typically reveals an ulcerated epidermis accompanied by a mixed inflammatory infiltrate composed of neutrophils, lymphocytes, and histiocytes, as well as small epithelioid granulomas with central necrosis, multinucleated giant cells, and Langhans cells in the dermis. Acid-fast bacilli (AFB) staining is positive in only a minority of histological specimens; thus, clinical, microbiological, and radiological correlation is crucial to ensure early and accurate diagnosis [25]. Rapid tests for diagnosing tuberculosis are accurate, cost-effective, and allow for rapid treatment initiation. Tuberculin skin testing (TST) and Interferon-Gamma Release Assay (IGRA) are used to detect latent TB. The latter is more specific and unaffected by BCG vaccination; nevertheless, it is more expensive. For active TB, smear microscopy is commonly used, but cultures are more sensitive and allow susceptibility testing. The TB-LAM test, with limited sensitivity, is useful in people with HIV and low CD4 counts. Molecular tests based on real-time PCR are rapid, sensitive, and specific, and they are recommended as the initial test for both pulmonary and extrapulmonary TB. The reported case showed positive results for Mtb on both bacilloscopy and culture.

AFB detection techniques include Ziehl–Neelsen, Fite–Faraco, and Auramine–Rhodamine stains. Likewise, Löwenstein–Jensen, a conventional egg-based solid medium, and automated broth systems, such as BACTEC 460 (Becton Dickinson Microbiology Systems) and the mycobacterial growth indicator tube, are the media with the fastest growth and are commonly used. The culture in the Löwenstein–Jensen medium, incubated at 37 °C, showed the growth of cream-colored colonies after 20 days of incubation and was compatible with mycobacteria. The Centers for Disease Control and Prevention recommends simultaneous use of a solid and liquid medium for culture [26].

The polymerase chain reaction (PCR) is a molecular biology technique that rapidly identifies Mtb, initiating prompt treatment. In addition, the GeneXpert MTB/RIF real-time PCR assay allows the detection of Mtb DNA and the detection of rifampicin resistance [27], facilitating early diagnosis. The quantiferon test is much more specific for Mtb than other mycobacterial subtypes [28]. Definitive diagnosis requires isolating the microorganism by culture or PCR sequencing since histological findings may be nonspecific but suggestive, considering that cultures are often reported negative. Consequently, diagnosis is sometimes based on clinicopathological correlation and response to anti-TB treatment.

Definitive diagnosis is based on bacteriological detection by bacilloscopy or culture. Sputum smear microscopy (Ziehl–Neelsen or fluorescence staining) is rapid but less sensitive. Culture remains the gold standard, as it detects viable bacilli in paucibacillary samples and allows for sensitivity testing and typing, although it is slower and more expensive. The LF-LAM (lateral flow lipoarabinomannan) test is useful in people with HIV with advanced disease, especially in hospitalized patients with CD4 < 200/mm^3^. Its sensitivity is moderate, but its specificity is high. Molecular methods have revolutionized TB diagnosis due to their rapidity and high sensitivity. These include Xpert MTB/RIF and Xpert-Ultra, which detect M. tuberculosis and rifampicin resistance in <2 h. Xpert MTB/XDR also identifies resistance to isoniazid and second-line drugs. Other rtPCR-based methods such as Truenat, BD MAX, Abbott, and Roche offer integrated tests with high throughput. LAMP allows for rapid detection of multidrug resistance, although it requires specialized laboratory conditions. TB-LAMP is a simple and low-cost alternative for primary care settings. Whole genome sequencing and targeted next-generation sequencing (NGS) allow for the identification of multiple resistance-associated mutations in a single assay and have been recommended by the WHO since 2024 [29].

Scrofuloderma is treated in the same way as a tuberculous disease in immunocompetent subjects. Treatment includes an initial 2-month phase with four antibiotics: rifampicin (10 mg/kg/day) + isoniazid (5 mg/kg/day) + pyrazinamide (20–30 mg/kg/day) + ethambutol (15–20 mg/kg/day), followed by a second phase with dual therapy with rifampicin and isoniazid. In HIV-positive patients, treatment duration is generally 6 months; nowadays, longer courses are sometimes recommended [30,31]. Even so, special attention should be paid to the presence of drug resistance since; in this case, treatment needs to be changed and extended. Drug doses are calculated according to the patient’s weight based on different protocols [32]. Likewise, an empirical anti-TB treatment could be initiated according to the clinical picture. Treatment of extensive scrofuloderma sometimes requires surgical intervention [33]. Finally, WHO has developed an operational manual on tuberculosis to provide practical guidance to complement its most recent guidelines on the treatment of drug-resistant tuberculosis. The document explains how to select and design treatment regimens for different types of drug-resistant tuberculosis, including multidrug-resistant or rifampicin-resistant tuberculosis (MDR/RR-TB) and isoniazid-resistant but rifampicin-sensitive tuberculosis (HR-TB) [34].

## 4. Conclusions

Scrofuloderma, a frequent presentation of CTB, may represent underlying bone, lymph node, joint, or testicular TB. Due to its clinical presentation, it is essential to consider and rule out differential diagnoses such as actinomycosis, syphilitic gumma, and pyoderma gangrenosum. A high index of clinical suspicion, coupled with an appropriate microbiological diagnostic strategy or molecular biology techniques, are critical for achieving timely treatment of this form of EPTB—an old but persistent clinical entity capable of causing severe sequelae and even death. In the present clinical case, an early diagnosis was achieved, accompanied by timely treatment, achieving a rapid resolution of the injury. Close monitoring is required to detect drug resistance or adverse events.

Tuberculosis prevention in people living with HIV is a fundamental health priority, given the high risk of progression from latent infection to active disease. Routine screening using TST or IGRA, along with clinical evaluation, allows for the identification of candidates for preventive therapy, even in the presence of immunosuppression. Prophylactic regimens, such as isoniazid or abbreviated combinations with rifapentine, have proven effective in reducing the incidence of active TB. Their implementation, along with early initiation of antiretroviral treatment, should be part of an integrated and sustained approach. The coordination of these strategies, supported by public policies and health system strengthening, is essential to reducing the disease burden in this vulnerable population and making progress toward global TB control goals.

## Data Availability

The original contributions presented in the study are included in the article, and further inquiries can be directed to the corresponding author/s.

## Abbreviations

The following abbreviations are used in this manuscript:

TB	Tuberculosis
EPTB	Extrapulmonary TB
CTB	Cutaneous TB
PCR	Polymerase Chain Reaction
